# NeuroVAD: Real-Time Voice Activity Detection from Non-Invasive Neuromagnetic Signals

**DOI:** 10.3390/s20082248

**Published:** 2020-04-16

**Authors:** Debadatta Dash, Paul Ferrari, Satwik Dutta, Jun Wang

**Affiliations:** 1 Electrical and Computer Engineering, University of Texas at Austin, Austin, TX 78712, USA; 2 Department of Neurology, University of Texas at Austin, Austin, TX 78712, USA; 3 Department of Psychology, University of Texas at Austin, Austin, TX 78712, USA; 4 MEG Lab, Dell Children’s Medical Center, Austin, TX 78723, USA; 5 Electrical Engineering, University of Texas at Dallas, Richardson, TX 75080, USA; 6 Communication Sciences and Disorders, University of Texas at Austin, Austin, TX 78712, USA

**Keywords:** brain-computer interface, MEG, wavelet, LSTM-RNN, SVM, VAD, speech-BCI

## Abstract

Neural speech decoding-driven brain-computer interface (BCI) or speech-BCI is a novel paradigm for exploring communication restoration for locked-in (fully paralyzed but aware) patients. Speech-BCIs aim to map a direct transformation from neural signals to text or speech, which has the potential for a higher communication rate than the current BCIs. Although recent progress has demonstrated the potential of speech-BCIs from either invasive or non-invasive neural signals, the majority of the systems developed so far still assume knowing the onset and offset of the speech utterances within the continuous neural recordings. This lack of real-time voice/speech activity detection (VAD) is a current obstacle for future applications of neural speech decoding wherein BCI users can have a continuous conversation with other speakers. To address this issue, in this study, we attempted to automatically detect the voice/speech activity directly from the neural signals recorded using magnetoencephalography (MEG). First, we classified the whole segments of pre-speech, speech, and post-speech in the neural signals using a support vector machine (SVM). Second, for continuous prediction, we used a long short-term memory-recurrent neural network (LSTM-RNN) to efficiently decode the voice activity at each time point via its sequential pattern-learning mechanism. Experimental results demonstrated the possibility of real-time VAD directly from the non-invasive neural signals with about 88% accuracy.

## 1. Introduction

Brain damage or late-stage amyotrophic lateral sclerosis (ALS) eventually leads to a stage of paralysis called locked-in syndrome, where the patients become completely paralyzed while being otherwise cognitively intact [1]. Assessing the neural pathways through brain-computer interface (BCI) may be the only approach to restore some level of communication for these patients [2,3]. Current EEG-based BCIs are primarily based on screen-based letter selection strategies using visual or attention correlates, resulting in a slow communication rate (a few words per minute) [4]. To address this issue, recent studies have explored direct mapping of neural signals to text (neural speech decoding) or speech acoustics (neural speech synthesis), which then drives a speech synthesizer. The speech decoding paradigm has the potential for faster, next-generation BCIs. Numerous studies have proven the feasibility of neural speech decoding [5,6,7,8,9]. For instance, recent studies have shown the possibility of phoneme, syllable, and word classification with electroencephalography (EEG) or electrocorticography (ECoG) and phrase classification with magnetoencephalography (MEG) [10,11,12]. Very recently, two studies on direct mapping of neural signals to speech with ECoG [13,14], have further strengthened the potential of neural speech decoding-driven BCIs.

One critical obstacle in developing neural speech decoding for future BCIs for dialog is the lack of real-time detection of voice/speech onset and offset. Most of the current work on neural speech decoding involve manual tagging of the speech activity segments within the neural recordings. To extend the research on speech-BCIs to real-world applications, entire decoding process has to be completely automated in real-time, including automatic detection of speech-motor activity segments in the neural signals. In other words, current experimental protocol of manual tagging of voice activity periods must be replaced with voice activity detection (VAD) systems, which can automatically identify the time points where the subject starts and ends speaking. Voice activity detection (VAD) has been widely used in automatic speech recognition (ASR), where the goal of VAD is to identify the onset and offset of voice/speech activities [15]. With VAD, modern ASR systems (e.g., Apple Siri) can have continuous conversations with users (speakers). Here, we borrowed the terminology ‘VAD’ and called voice activity detection from neural signals as ‘NeuroVAD’. The assumption is that the neural activity patterns during speech would be different from those during silence, such that a VAD system (NeuroVAD) can be implemented to automatically determine the initiation of speech-motor activity in the neural signal and then fed to a speech-BCI for decoding. Very recently, a few studies have attempted this problem with invasive ECoG [16,17]. The former study with ECoG involved word-level VAD (picture naming task) and the later was at the syllable-level. Although these studies are promising, to our knowledge, no prior study has reported identifying the voice onset and offset based on neural signals while a subject is producing continuous phrases, which is attempted here.

In this study, we used MEG to record the neural signals while 8 subjects produced 5 phrases. MEG records the magnetic fields corresponding to post-synaptic neuronal currents from the cortical surface. It has a high spatial (higher than EEG) and temporal (higher than fMRI) resolution. The advantages of high temporal resolution are particularly important for neural speech-motor studies considering the fast and subtle dynamics of speech production. More importantly, MEG is non-invasive (in contrast to ECoG), hence, more practical for wider applications. Furthermore, recent research on MEG-based speech studies [18,19] suggest that it can capture the fast dynamics of neural speech characteristics. Although the current MEG machine is limited by non-portability, cost, and size; recent studies with optically pumped magnetometers (OPM) [20] are certainly promising towards overcoming these limitations.

The major assumption for VAD from neural signals is that the neural activities corresponding to speech and silence are different. Although previous research [17] has shown the possibility of isolated classification of ’speech vs. non-speech’ using invasive (ECoG) neural signals, no published research has been done using non-invasive neural signals for real-time VAD to our knowledge. We first classified the whole segment of speech versus non-speech from the neural signals using a support vector machine. Second, considering the advantage of long short-term memory-recurrent neural network (LSTM-RNN) for pattern-learning in non-stationary time series signals, we used LSTM-RNN to detect real-time voice activity from the MEG signals.

## 2. MEG Methods

### 2.1. Data Collection

**Figure 1:**
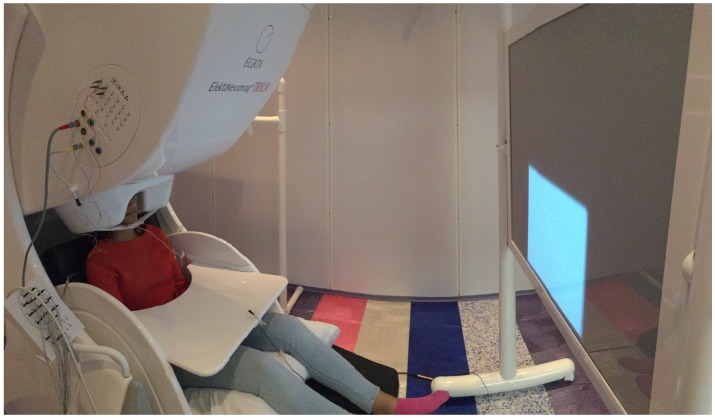
The MEG unit.

Two identical Triux Neuromag MEG devices (MEGIN, LCC) were used for data collection from eight subjects, one at Cook Children’s Hospital, Fort Worth, Texas (Figure 1) and the other at Dell Children’s Medical Center, Austin, Texas. The MEG machines consist of 306 channels with 204 planar gradiometer sensors and 102 magnetometer sensors and are housed within a magnetically shielded room (MSR) to discard external magnetic field interference. MEG data were recorded with a sample rate of 4 kHz and an online filter of 0.3 to 1 kHz. Vocal responses were recorded directly into the MEG systems’ ADC channels using a standard microphone with the transducer situated outside the MSR. Movement of the jaw was also recorded on an ADC channel via a custom build pressure sensor attached to an air bladder placed on the chin. Prior to experimentation, the subjects’ head coordinate system based on three fiducial points was created for the subjects using the FastTrak digitization system (Polhemus Ltd.). Five head-position-coils placed on the scalp were digitized to facilitate head localization within the MEG helmet. Bipolar electrooculography (EOG) and electrocardiograph (ECG) sensors were used for recording the eye movement and cardiac signals, respectively.

### 2.2. Participants and Stimuli

Although the ultimate goal of this research is targeted for locked-in patients, it is essential to first learn whether a NeuroVAD system is possible for healthy subjects before moving on to ALS patients. Hence, to show the first proof of concept of NeuroVAD with non-invasive neural signals, in this study we used data from 8 healthy subjects (3 females and 5 males; age 41 ± 14 years) without a history of vision, speech, and auditory or cognitive disorder. Prior written consent was obtained from each subject in compliance with the institutional review boards of UT Dallas, UT Austin, Dell Children’s Medical Center, and Cook Children’s Medical Center. A set of 5 commonly used phrases used in augmentative and alternative communication (AAC) were chosen as the stimuli for this study. They are: *1. Do you understand me, 2. That’s perfect, 3. How are you, 4. Good-bye, and 5. I need help*. A stimulus dedicated computer running the STIM2 software (Compumedics, LTD) and connected to a high-quality DLP projector was used to visually display the phrase stimuli onto a back-projection screen situated at about 90 cm from the subjects.

### 2.3. Protocol

**Figure 2:**
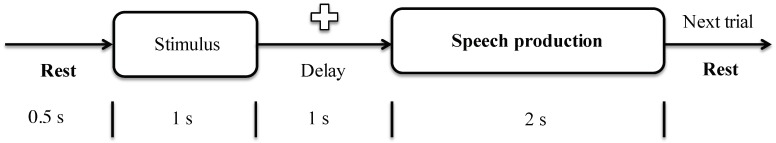
Time-locked protocol.

Subjects were seated comfortably in the MEG chair with their head positioned carefully inside the MEG helmet. The protocol was designed as a time-locked delayed overt reading task as shown in Figure 2. The first 500 ms prior to stimulus onset served as baseline. Then, the phrase stimulus was displayed for 1 second on the screen. It was followed by a delay of 1 second where the stimulus was replaced by a fixation cross (+) heralding the subjects to imagine and prepare for the articulation of the previously shown phrase. The removal of the fixation (blank screen) cued the subjects to articulate the previously shown stimulus at their natural speaking rate. This protocol was repeated for 100 trials per stimulus per subject in a pseudo-randomized order following the standard practice of MEG experiments [21,22]. Subjects were first trained with some sample stimuli for compliance. The whole experiment per subject lasted for an average of 45 min.

### 2.4. Data Preprocessing

All data were epoched into trials from −0.5 s to 4.5 s, with respect to stimulus onset, using MATLAB code from standard toolboxes. Through visual inspection, high amplitude artifacts and untimely articulated trials were discarded from analysis. For example, occasionally subjects would create very large jaw clenching, or they would speak too late and that would carry on to the next trials. Those trials were removed. A total of 3046 valid trials were retained after preprocessing from 4000 (8 *subjects* × 5 *phrases* × 100 *trials*) experimental trials with an average of 75 trials per phrase per subject. Only gradiometer sensors were considered in this study considering their effectiveness in noise suppression. Noisy or unresponsive channels were removed from analysis. The number of sensors were roughly equivalent across machines and subjects. Out of 204 gradiometer sensors, on average only 200 sensors in both the MEG machines were used for further analysis. Based on the effectiveness of wavelets in denoising the MEG signals [23], a 2 level Daubechies (db)-4 discrete wavelet decomposition was performed to denoise and restrict the signals up to high-gamma frequency (<125 Hz). For subsequent processing and analysis, only a 2 second period starting at the speech production cue was forwarded for analysis.

### 2.5. Data Labeling

**Figure 3:**
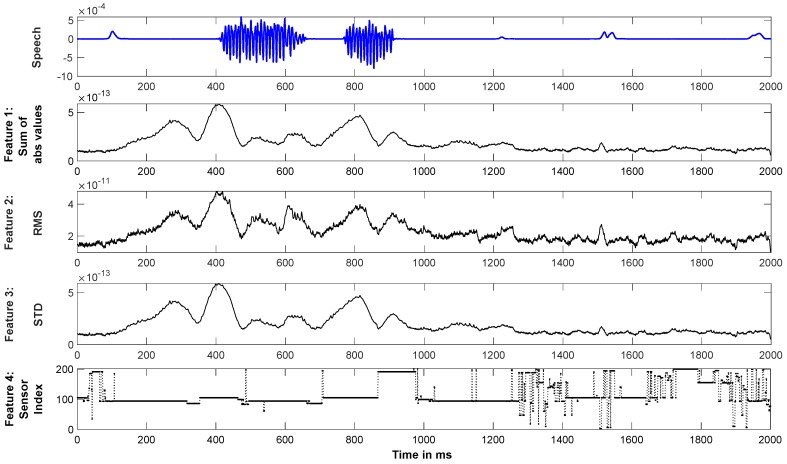
The four features selected for the experiment are shown for one example trial (in black). Corresponding speech signal is shown in blue. Feature 1: sum of absolute (abs) values; Feature 2: root mean square (RMS) values; Feature 3: standard deviation (STD) across sensors; Feature 4: Index of the sensor with highest magnitude at each ms of time.

For obtaining the ground truth, each sample of data within the 2 s time window of analysis was labeled as ‘speech’ or ‘non-speech’. The recorded speech signals were noisy and hence were first denoised with Wiener filtering [24]. The time segment starting from the cue of articulation until the time when the subject starts speaking, as indexed by the onset of the acoustic voice signal, was labeled as ‘Pre-Speech’ (Pre). The time interval where the subject was speaking (producing acoustic output) was labeled as ‘Speech’, and the remaining time interval, i.e., after ending of articulation to the end of production segment (within the 2 s stage of speech production stage) was labeled as ‘Post-Speech’ (Post). For example, from Figure 3, the subject started speaking at 401 ms after the cue and ended at 931 ms. In this case, the time interval from 1 ms to 401 ms was labeled as ‘Pre-Speech’, 402 to 931 ms as ‘Speech’, and the period between 932 to 2000 ms (end of articulation) as ‘Post-Speech’. This three-category labeling was done for an ‘Isolated Classification’ analysis (see below). For real-time prediction, time points were labeled as either ‘Speech’ or ‘Non-Speech’, with ‘Post-Speech’ segments being collapsed into the ‘Non-Speech’ category. In the Figure 3 example, all the time points from 1 ms to 401 ms and from 932 ms to 2000 ms were labeled as ‘Non-Speech’, and those from 402 ms to 931 ms as ‘Speech’.

## 3. Decoding Methods

The objective of this study is to perform voice activity detection in neural signals, essentially to determine the start and the end time point of speech-motor activity from the MEG signals, such that it can be isolated and fed to a speech-BCI. For this, first, we performed a sanity check to see if there is a difference between speech and silence segments within the neural signals. We implemented this using a support vector machine classifier. The details are mentioned in the ‘Isolated Classification’ section below. After getting a significant classification performance, we proceeded toward building the NeuroVAD algorithm for continuous prediction. For this, we extracted features from the MEG sensor signals at each time point (see details below). Then those features were trained using LSTM-RNN to leverage the dynamic temporal information of the brain signals. In this experiment, as in previous research, we observed high cognitive-performance variance across subjects, hence we focused this work on subject-dependent prediction [25]. Also, data from the whole head sensors were used for the prediction analysis because it has been previously shown that the whole head information outperforms that using only the speech center region sensors in neural speech decoding [9].

### 3.1. Isolated Classification

We used the root mean square (RMS) features of the MEG signals to classify the brain activity signals corresponding to speech and silence, as RMS feature has been proven to be effective in previous MEG studies [7,26]. RMS features obtained from speech and non-speech segments were fed to a binary 2nd order polynomial kernel SVM classifier. SVM was chosen as the choice of classifier because of its efficacy with multi-dimensional data (here 200-dimensional RMS features from 200 gradiometers). Furthermore, to obtain a more significant information about the difference between neural behavior among ‘Pre-Speech’, ‘Post-Speech’, and during Speech, we performed multiple classifications between these segments such as ‘Pre-Speech’ v ‘Post-Speech’, ‘Pre-Speech’ v ‘Speech’, ‘Post-Speech’ v ‘Speech’, and ‘Pre-Speech’ v ‘Speech’ v ‘Post-Speech’ (3 class classification with ‘one v one’ scheme). A 6-fold cross validation strategy was implemented for all the classification tasks.

### 3.2. Continuous Prediction

#### 3.2.1. Feature Extraction

After verifying, via isolated classification, that speech and non-speech segments could be predicted using a magnitude-based statistical feature, we proceeded under the assumption that speech-related sensors will have selectively higher amplitudes. To leverage the temporal dynamics of the MEG signal, we extracted four feature sets at each time point across all the sensors to train the LSTM-RNN deep learning algorithm for continuous temporal prediction of ‘Speech’ vs. ‘Non-Speech’. The first three features were the ‘absolute magnitude’, ’root-mean-square’ (RMS), and ’standard deviation’ which have been shown to be effective in previous studies [7,26,27,28]. The 4th feature was the index of the most active sensor, i.e., the sensor with maximum magnitude value among all sensors at each time point. This feature inserted spatial information into the algorithm under the assumption that sensors overlying speech-related brain regions would be more consistently active during speech. Other features such as cross-correlation value, windowed energy, frequency distribution, and Shannon entropy were also evaluated, but the said four features were found to provide the best validation accuracy and hence were chosen as the final feature set. Figure 3 shows the temporal profile of the four features plotted against a corresponding speech signal.

#### 3.2.2. LSTM-RNN

LSTM-RNN is a sequential deep learning model which can continuously predict the probability of classes at each time point on sequential data [29]. LSTMs were developed to deal with the vanishing gradient problem that was encountered with conventional RNNs [30]. LSTM is argued to be the most significant commercial achievement in deep learning, which is used mostly for time series data [29]. Since MEG signals have very fast temporal dynamics, use of LSTM-RNN was the logical choice for this study. Also, LSTMs have been recently proven to be effective in decoding speech stages with MEG signals [18]. Our model consisted of two hidden layers each with 256 nodes. The hidden layers contained the learnable parameters in the form of input and recurrent weights. The layers are typically designed as a vertical concatenation of the input/recurrent weight matrices for the components (gates) of the LSTM layer namely input gate, forget gate, cell candidate, and output gate in respective order. The gate state was updated with a hard-sigmoid activation function. The tanh activation function was used to update the cell and hidden states. This specific arrangement of memory blocks has been shown to help in performing additive interactions to improve gradient flow over long sequences during training [31].

The hidden LSTM layer was then followed by a fully connected layer and a SoftMax layer, each with 2 nodes to decode the class information (‘Speech’/‘Non-Speech’) via simultaneously updating the network states. The network was trained with back-propagation through time (BPTT) algorithm via an Adam optimizer with a gradient threshold of 0.1, gradient threshold method of *L*_2_ norm, and a maximum epoch of 500. The maximum number of epochs was decided based on the validation performance to check data overfitting. 50 trials of the data (of each phrase stimulus) were used for training and the remaining trials were used for testing. Hyper-parameters such as initial learning rate (=0.004 − 0.006), number of layers (=2), number of hidden units (=256), beta1 (=0.9), beta2 (=0.998), epsilon (=10*E* − 8), were chosen based on the hyperparameter search for best validation results.

## 4. Results and Discussions

### 4.1. Isolated ‘Speech’ vs. ‘Non-Speech’ Classification

Human brain activity during speech and silence has been studied extensively in past decades to obtain a better understanding of the neural mechanism of speech production [32,33]. However, the ‘speech’ tasks in many of these studies had been designed to evaluate either non-speech acoustic production or various oro-motor tasks that may not represent speech production in its entirety. Little is known about the neural behavior in pre-speech or post-speech intervals during overt speech production. The pre-speech neural data consists of the reaction time as well as the speech-motor initiation prior to acoustic output. Hence, brain activity during the ‘Pre-Speech’ and ‘Speech’ segments might have significant overlap, with the addition of auditory feedback for the ‘Speech’ segment. Similarly, with ‘Post-Speech’, the auditory and language related cortex process information long after the speech acoustics terminate [34]. Hence, determining the beginning and end of articulation solely from brain activity may also be challenging. In this study, an SVM classifier was tasked with these objectives.

**Figure 4:**
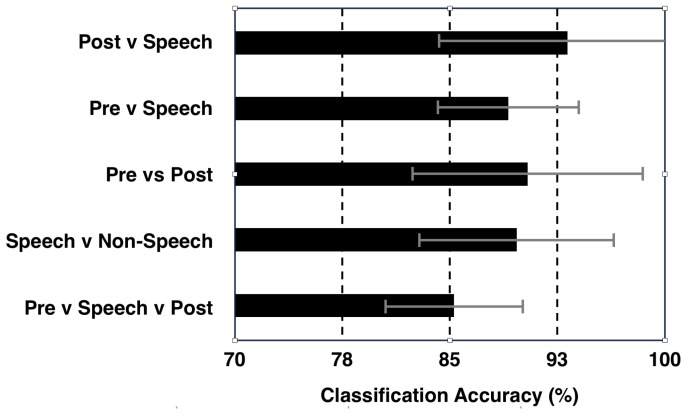
Results of classification between Pre-, Post-, and Speech segments; Error bars show the standard deviation across 8 subjects).

Figure 4 shows the isolated classification results obtained with the SVM classifier averaged over 8 subjects. A 90% classification accuracy was observed between ‘Speech’ and ‘Non-Speech’ (‘Pre-Speech’ + ‘Post-Speech’) classes. This result provides compelling evidence that differences in the brain signals between speech and silent intervals can be differentiated and used for automated detection of speech onset and offset, and thus providing motivation for developing a NeuroVAD algorithm. Additional classifications were performed by separating the ‘Pre-Speech’ and ‘Post-Speech’ segments into their own classes. Results for all classification combinations are shown in Figure 4. A 3-class classification for all segments resulted in approximately 85% accuracy, indicating that two of the 3 classes contained redundant information. In comparing classification of ‘Speech’ against ‘Pre-’ or ‘Post-Speech’, the ‘Speech’ vs. ‘Post-Speech’ resulted in higher accuracy. This again highlights the difference between ‘Pre-’ and ‘Post-Speech’ segments. Considering the feature sets for the LSTM-RNN model, it is clear that there is a considerable overlap of feature shapes—of low-frequency modulation—across ‘Pre-Speech’ and ‘Speech’ segments, whereas the ‘Post-Speech’ segment demonstrates fairly low variance. From our movement data we know that oromotor movement precedes acoustic onset, which may explain this. Similarly, Feature 4 seems to mirror this differentiation with the dominant sensor location being more stable over low-frequency modulation segments of ‘Pre-Speech’ and ‘Speech’ and more variable as they subside in ‘Post-Speech’. This could mean that Feature 4 tracks the low-dimensional transitions of brain network formation for specific tasks. While this observation generates interesting speculation, the statistical difference between these decoding schemes were not significant (‘Pre-Speech’—‘Speech’ vs. ‘Post-Speech’—‘Speech’, *p* = 0.164; 1-tailed *t*-test; 95% CI). This may be due to one outlier subject who was the only subject to show better ‘Pre-Speech’ vs. ‘Speech’ classification. This indicates that the study is most likely underpowered for such statistical comparisons. Regardless, the high classification accuracy (>89%) of speech interval compared to ‘Non-Speech’ (both ‘Pre-Speech’ and ‘Post-Speech’ segments) further boosts confidence for designing an automated NeuroVAD system.

### 4.2. Real-Time NeuroVAD

**Figure 5:**
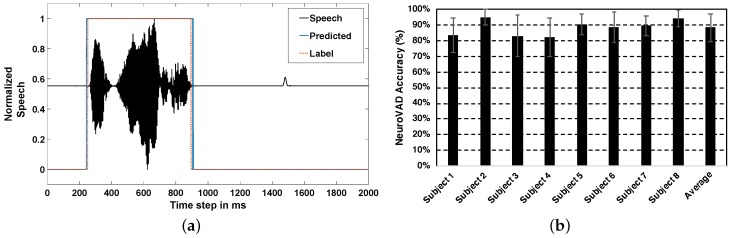
Prediction results with NeuroVAD: (**a**) while subject 8 was speaking “That’s perfect” and (**b**) Prediction accuracy with LSTM-RNN for all 8 subjects and their average; Error bars indicate the standard deviation in predictions across trials.

The result of the automatic neural voice activity detection experiments can be observed in Figure 5, which shows an exemplary prediction result plotted over the corresponding speech data. The speech signal in Figure 5a is when subject 8 was speaking “That’s perfect”. The red dotted line indicates the ground truth labeling and the blue dotted lines indicate the prediction by LSTM-RNN. Since the objective was just to find the start and endpoints of a phrase, the silences within a phrase were not designed to be predicted. The predicted start time points were slightly earlier (about a mean of 10 ms) than the truth (labels), which was common for most of the test trials; however, no statistical significance was found for this result with left tailed *t*-test (*p* = 0.068). Despite the prediction error and theoretical decrease in the computational accuracy, one interpretation of this behavior is that the neural information corresponding to speech might occur before the actual speech production, as illustrated in Figure 3, where an increase in the magnitude of feature values from the baseline can be observed slightly before the speech onset. Future analysis, however, is needed to confirm this interpretation. Nevertheless, the predictions are accurate, indicating that the start and end time points are determinable from the neural signals alone.

The overall performance results of the NeuroVAD experiments are shown in Figure 5b which represents the accuracy of NeuroVAD in single-trial levels for each subject and their average. Here, accuracy = (TP + TN)/(TP + TN + FP + FN); where: TP = true positive; FP = false positive; TN = true negative; FN = false negative. The average accuracy was found to be 88.07% across 8 subjects. The classification accuracy reported in [17] was about 92%, using isolated classification to differentiate between ‘Speech’ and ‘Non-Speech’. This is similar to our method (see Figure 4) which classifies with about 90% accuracy. Going further, our result with LSTM-RNN is for predicting ‘Speech’ labels at each time point (real-time) as is done in conventional VAD systems in ASR community. Continuous prediction is crucial for speech-BCI applications for designing conversational systems similar to Alexa or Siri but with neural signals instead of speech signals for ALS patients. Although the accuracy obtained was promising, conventional VAD systems have near-perfect accuracy in current speech assistant technologies. Hence, more research is required to increase NeuroVAD performance. One possible solution is to use various deep learning feature extractors instead of the statistical features used in this study; however, an exponentially larger dataset would be required. It is very difficult to collect a lot of neural data, specifically for the ALS population, the patients get tired eventually during the task, considering the higher amount of motor involvement to compensate for the paralyzed muscles [35]. Thus, intelligent strategies such as data augmentation or artificial data synthesis and transfer learning techniques might be needed for effective decoding. Also, the machine training strategy has to be optimized in a way that the training and validation should be performed with one session data and testing should be done with the next session data, making the algorithm more robust and invariant across sessions for ALS patients [36,37]. Of note that our data preprocessing step involved discarding of artifact biased trials through visual inspection leading to a 25% rejection rate. This might be very costly in the low data regime of ALS population, and thus, instead of trial rejection, data denoising strategies should be implemented [38].

**Figure 6:**
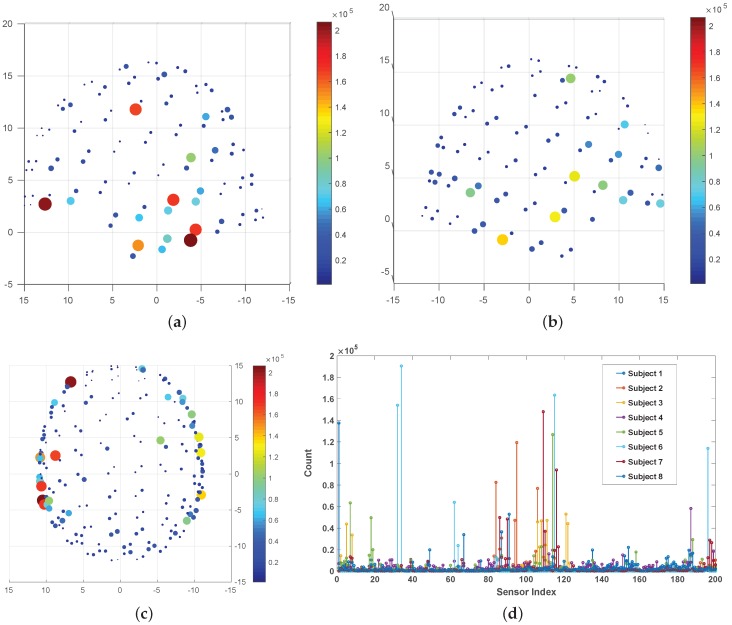
Frequency plot of most active sensors across all 8 subjects shown with 3 views: (**a**) left (**b**) right, and (**c**) axial. Each dot represents a sensor. Color and size represent the count. (**d**) represents a histogram plot of the frequency (count) of all the active sensors for each subject individually represented by a unique color.

In Figure 5b, it can be observed that the accuracies are somewhat subject specific, possibly, due to the inclusion of all the sensors (whole head) in the feature set. It is challenging to determine the exact combination of sensors that will provide the best performance. Also, intersubject cognitive-performance variance might also have played a role in prediction variance. A statistical frequency (mode) analysis of the sensor indices across all 8 subjects yielded commonalities in sensors in the temporal regions bilaterally with a bias to the left hemisphere Figure 6a–c. This is consistent with the traditional understanding of left hemisphere dominance of speech production in neuroanatomy. While this analysis highlights common sensors across subjects, there is still large intersubject variability as shown in Figure 6d. This variability may speak to the high level of inter-individual variation in neuro-cognitive network structure observed in neuroimaging experiments and reflect the necessity of subject-dependent decoding [39]. Nevertheless, the efficacy of this study is in getting high accuracy in single-trial level with an average standard deviation of <10%. For all the subjects, the median was higher than the mean, with the overall distribution within 85–90%. However, there were a few trials for which the accuracy was not very satisfactory (70–80%). Since the accuracy was calculated in the sample level of single-trials, a 10% misclassification in one trial corresponds to about 200 samples being misclassified. However, the recall of this study across all subjects (92.33%) was higher than the precision (90.92%) indicating fewer false negatives (actual ’Speech’ being predicted as ’Non-Speech’) than false positives. It is also important to mention that most of the false positives were near the boundary where the NeuroVAD algorithm mispredicted the ’Non-Speech’ labeled samples those were just before and after the acoustic onset and offset respectively as ’Speech’. Although it reduces the machine accuracy, this result can be reasoned as the outcome of pre-cognitive planning and post-auditory processing in the brain for speech production, which might increase the BCI performance if included.

### 4.3. Efficacy of LSTM-RNN for NeuroVAD

**Figure 7:**
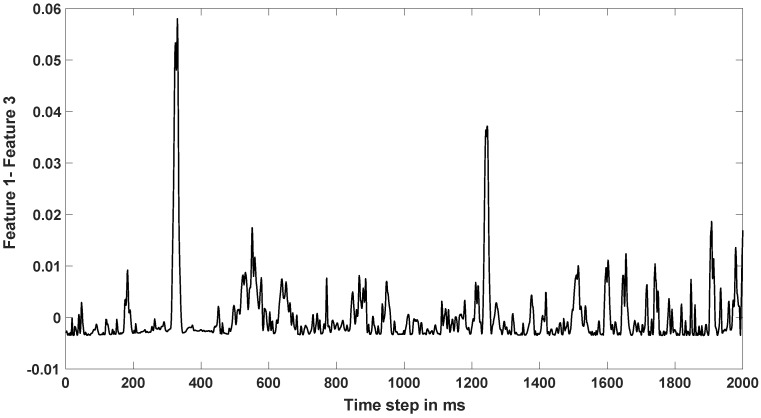
Difference between Feature 1 (Sum of absolute values) and Feature 3 (Standard deviation) for one example trial.

In regards to the performance of LSTM-RNN, it is worth mentioning that it was capable of learning the sequential pattern from the features corresponding to speech and non-speech periods. One point of discussion is with the features used for training the LSTM-RNN. The three statistical features used here, especially Feature 1 (sum of absolute values) and Feature 3 (standard deviation) were correlated. However, discarding any of these 3 features from the feature set resulted in about 5% decrease in accuracy. To further analyze, we plotted the difference between these 2 features (Feature 1 and 3) as shown in Figure 7. Although Feature 1 and 3 seems similar in Figure 3, they have differences in time and thus both the features were required and contributed to NeuroVAD training. We also implemented various combinations of time frequency representational features as proposed in [17] but the validation accuracy for NeuroVAD was optimal with the current 4 statistical features. Rigorous hyperparameter tuning was performed to find the best architecture for this objective. To mention a few, around a 4% increase in accuracy was obtained when the number of layers increased from one to two and then saturated with more layers. Similarly, there was around a 3% increase on average accuracy when the number of nodes increased from 64 to 128 and then 256, beyond which accuracy saturated. The addition of another fully connected layer after the LSTM layer did not improve accuracy. Overall, the proposed architecture was found to provide the best performance in voice activity detection and LSTM-RNN was found to be efficient for this objective. As this NeuroVAD approach is constructed to learn the features in the temporal domain, and considering both EEG and ECoG reflect similar data types, a major contribution of this study is that the proposed approach can be translated to the EEG and ECoG modalities.

### 4.4. Future Work

To establish the proof of concept for a non-invasive NeuroVAD system, our starting point was to use data from healthy subjects. However, it is unknown whether the neural activity (during speech or silence) of ALS patients would be the same as the healthy subjects. Therefore, future work will investigate this approach with data from ALS patients for speech-BCI applications. While it is uncertain that the same features or architecture will result in equivalent performance, the proposed approach can be easily modified for intelligent feature selection from neural data of the ALS brain. Furthermore, the NeuroVAD is purposefully designed to be subject-dependent owing to the high cognitive-performance variance even within healthy subjects. Future studies will aim at identifying the source of this variance in an effort to improve classification and possibly more toward subject independent applications.

## 5. Conclusions

In this study, we investigated the automatic, real-time detection of voice activity from neural (MEG) signals (NeuroVAD) using LSTM-RNN. First, we classified the speech and silence intervals from the MEG signals via an SVM classifier with 90% accuracy which indicated that the prediction of start and end time points of speech-motor activity is possible. Furthermore, a NeuroVAD algorithm was developed using LSTM-RNN for the automatic real-time detection of voice onset and offset from neural signals. An average of about 88% accuracy was obtained in the single-trial level. With this high performance, NeuroVAD has the potential to be used as a necessary prerequisite to future speech-BCIs for continuous conversations, thereby showing promise to extend lab research into real-world applications. Moreover, the observation regarding machine predicted voice onset to be slightly before the acoustic speech onset, motivates for further research to better understand the temporal patterns in neural processing for speech production.
